# Risk of periprosthetic fracture after anterior femoral notching: A 9-year follow-up of 200 total knee arthroplasties

**DOI:** 10.3109/17453670903350099

**Published:** 2009-10-01

**Authors:** Narendra Gujarathi, Amit B Putti, Rami J Abboud, James G B MacLean, Arthur J Espley, Catherine F Kellett

**Affiliations:** ^1^University Department of Orthopaedics, Ninewells HospitalDundeeUK; ^2^Perth Royal InfirmaryPerthUK

## Abstract

**Background** Notching of the anterior femoral cortex in distal femoral fractures following TKR has been observed clinically and studied biomechanically. It has been hypothesized that femoral notching weakens the cortex of the femur, which can predispose to femoral fractures in the early postoperative period. We examined the relationship between notching of the anterior femoral cortex during total knee replacement (TKR) and supracondylar fracture.

**Patients and methods** Postoperative lateral radiographs of 200 TKRs were reviewed at an average of 9 (6–15) years postoperatively. 72 knees (41%) showed notching of the anterior femoral cortex. Notches were classified into 4 grades using the Tayside classification as follows. Grade I: violation of the outer table of the anterior femoral cortex; grade II: violation of the outer and the inner table of the anterior femoral cortex; grade III: violation up to 25% of the medullary canal (from the inner table to the center of the medullary canal); grade IV: violation up to 50% of the medullary canal (from the inner table to the center of the medullary canal) and unclassifiable.

**Results** The interobserver variability of the classification system using Cohen's Kappa score was found to be substantially reliable. 3 of the 200 TKRs sustained later supracondylar fractures. One of these patients had grade II femoral notching and the other 2 showed no notching. The patient with femoral notching sustained a supracondylar fracture of the femur following a simple fall at home 9 years after TKR.

**Interpretation** There is no relationship between minimal anterior femoral notching and supracondylar fracture of the femur in TKR.

## Introduction

Periprosthetic fracture after total knee replacement (TKR) has a reported incidence of between 0.3% and 2.5% (Delport et al. 1984, Wiedel 1984, Webster and Murray 1985, Ritter et al. 1988, Huo and Sculco 1990). Documented risk factors are osteoporosis, osteopenia, rheumatoid arthritis, neurological disorders, chronic steroid treatment and anterior femoral notching (Cain et al. 1986, Aaron and Scott 1987, Madsen et al. 1989, Huo and Sculco 1990). The possible role of notching of the anterior femoral cortex in distal femoral fractures following TKR has been observed clinically and studied biomechanically (Cain et al. 1986, Aaron and Scott 1987, Culp et al. 1987, Healy et al. 1993, Lesh et al. 2000). Hirsh et al. (1981) hypothesized that femoral notching weakens the cortex of the femur, which can predispose to femoral fractures in the early postoperative period. The incidence of supracondylar fracture in a notched femur following TKR varies from 0.5% to 52% (Hirsh et al. 1981, Aaron and Scott 1987, Culp et al. 1987, Ritter et al. 1988, Scott 1988, Bogoch et al. 1988, Madsen et al. 1989, Figgie et al. 1990, Booth 1994). Culp et al. (1987) studied 61 supracondylar fractures following TKR. They found that 27 cases showed notching of the anterior femoral cortex that had occurred during the surgery. 17 patients were suffering from a neurological disorder and the remainder had either rheumatoid arthritis or osteopenia. Lesh et al. (2000) investigated the biomechanical effects of notching of the distal anterior femoral cortex in TKR using human cadaveric femora, and found a mean reduction of 18% in bending strength and a 42% mean decrease in torsional strength for full-thickness notching of the femur. Zalzal et al. (2006) studied a 3-dimensional finite element model of the femur under gait loads and found that anterior femoral notches greater than 3 mm with sharp corners located directly at the proximal end of the prosthesis produced the highest stress concentrations and may lead to a substantial risk of periprosthetic fracture. However, a direct relationship between these events has not been established in the literature. A grading system for anterior femoral notching does not exist. The aims of this study were firstly to evaluate the incidence of anterior femoral notching in TKR, secondly to classify notching radiographically according to the degree of involvement of the anterior femoral cortex and medullary cavity, and also to assess interobserver variability. In addition, we wanted to study the incidence of supracondylar fracture after TKR in relation to anterior femoral notching.

## Patients and methods

We performed a retrospective study of 200 TKRs in 155 patients who were operated between 1984 and 1993. There were 98 women (63%) and 57 men (37%) with a mean age of 69 (23–88) years. 45 patients had bilateral procedures. Postoperative true lateral radiographs of 200 total knee replacements were reviewed at an average of 9 (range 6–15) years. Following scrutiny of these true lateral radiographs (taken immediately postoperatively or taken within 3 months to 1 year if the original radiograph was not a true lateral one), patients were divided into 3 major groups: notched, no notch, and unclassifiable. The patients for whom there were no records available or inadequate lateral radiographs were included in the unclassifiable group. These 3 major groups were divided into 2 subgroups according to the type of prosthesis used, either Kinematic condylar (Howmedica, Rutherford, NJ) (92 knees, 1984–1990) or Kinemax (Howmedica) (108 knees, 1990–1993).

**Figure 1.**
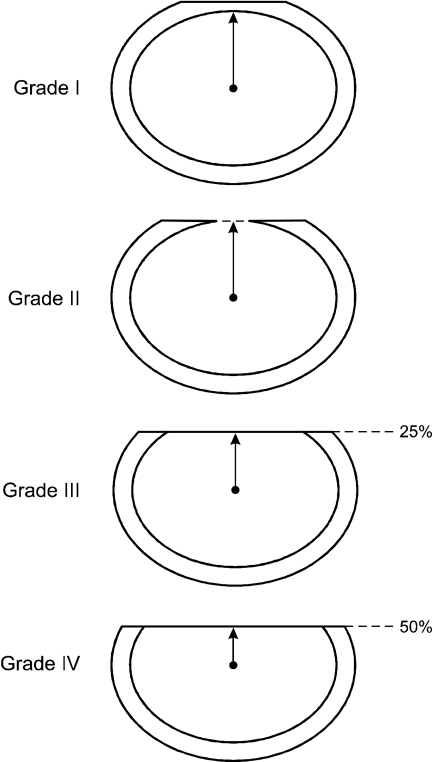
Transverse sections of the distal femur showing notching.

**Figure 2.**
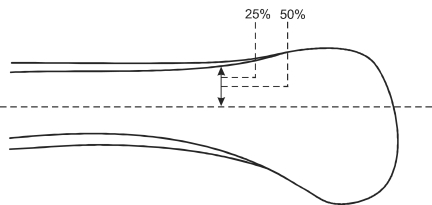
Sagittal section of the distal femur demonstrating grade III and grade IV notching.

In this study, notching was classified into 4 grades as follows (Figures 1 and 2): grade I: violation of the outer table of the anterior femoral cortex; grade II: violation of the outer and the inner table of the femoral cortex; grade III: violation up to 25% of the medullary canal (from the inner table to the center of the medullary canal); grade IV: violation up to 50% of the medullary canal (from the inner table to the center of the medullary canal). The 200 knees were classified as having no notch, a grade I to grade IV notch, or unclassifiable.

As validation for this new scoring system, interobserver error was assessed. 60 patients with 78 TKRs were randomly selected. The best true lateral radiograph (taken immediately postoperatively or up to 1 year) was selected, marked, and examined by 4 observers independently. Cohen's Kappa score was used to assess the variability in the finding. Once the grading was validated, the null hypothesis that notching of the anterior femoral cortex does not increase the risk of supracondylar fractures in total knee replacement was tested.

## Results

Notching of the anterior femur during TKR was observed in 72 of 176 radiographs (41%). Of these 72 notched knees, 35 were with Kinematic condylar knee prosthesis and 37 were with Kinemax knee prosthesis. The number of knees without notching was 102, and 26 knees were unclassifiable. The median interobserver reliability Kappa coefficient for this classification system using 4 observers was 0.74 (0.65–0.84), i.e. substantially reliable (Landis and Koch 1977). 3 patients in this series sustained a supracondylar fracture of the femur, with an incidence of 1.5%. 2 of these patients had rheumatoid arthritis but no notching, and one patient who had osteoarthritis had grade II notching of the anterior femoral cortex. The patient with the notched knee developed an undisplaced supracondylar fracture of the femur 9 years after surgery, caused by a fall at home. The fracture was treated by applying a cylindrical cast for 4 weeks followed by a functional cast brace for 6 weeks, after which the fracture had united. In the 2 patients without notching, one developed a displaced supracondylar fracture 6 months after surgery and the other patient developed an undisplaced supracondylar fracture of the femur 9 months after surgery. Both of these cases had rheumatoid arthritis. The null hypothesis that notching of the anterior femoral cortex does not increase the risk of supracondylar fracture in total knee replacement was accepted (p = 0.8, Student's t-test).

## Discussion

During the preparation of the distal femur, notching is relatively common despite sophisticated instrumentation. In the literature, the incidence of notching in TKR has been observed to be as high as 30% (Ritter et al. 2005). The incidence of notching of the anterior femoral cortex in TKR in this series was 41%.

Culp et al. (1987) measured the depth of notching on the lateral postoperative radiographs. They concluded that violation of the anterior femoral cortex in the supracondylar region up to 3 mm reduces its torsional strength by 29%. However, the depth of encroachment of anterior femoral cortex and medullary cavity of the distal femur has not been classified clinically or radiographically in the literature. In our study, the depth of the notching was classified into 4 grades according to the encroachment of the femoral cortex (outer and inner table) and medullary canal. The validity of this new classification for notching was substantially reliable.

The reported incidence of supracondylar fracture of the femur after TKR has ranged from 0.3% to 2.5% (Delport et al. 1984, Wiedel 1984, Webster and Murray 1985, Ritter et al. 1988, 2005, Huo and Sculco 1990). The incidence of supracondylar fracture of the femur in TKR with anterior femoral notching varies from 0.5% to 52% (Hirsh et al. 1981, Aaron and Scott 1987, Culp et al. 1987, Bogoch et al. 1988, Ritter et al. 1988, Scott 1988, Madsen et al. 1989, Figgie et al. 1990, Booth 1994). In our study, of the 72 notched femurs, supracondylar fracture was observed in only one patient (1.4%). This patient sustained a supracondylar fracture of the femur 9 years after surgery following a fall at home. The Tayside femoral notching grade was II. This suggests there is no direct relationship between notching and supracondylar fracture of the femur after TKR in our series. The other 2 patients, with no notching, developed supracondylar fractures of the femur 6 months and 9 months after their TKR and they had rheumatoid arthritis, which is associated with poor bone quality. The literature has shown that various factors contribute to the pathogenesis of supracondylar fracture of the femur after TKR such as the relative difference in elastic modulus between the femoral cortex and the metal prosthesis, stress shielding caused by the anterior femoral flange at bone-metal junction, postoperative hypovascularity leading to inadequate osseous remodeling, osteolysis caused by polyethylene wear debris (Rand 1994), and bone cement or metal causing endosteal ischemia (Short et al. 1981, Ritter et al. 1988). A combination of the axial and torsional loads plays an important role in the mechanism of these fractures (Insall 1984, Culp et al. 1987). The majority of the fractures occur after a simple fall. The other reasons for this type of fracture are manipulation of a stiff knee after TKR (Sisto et al. 1985), patients with seizures, or patients involved in motor vehicle accidents after TKR.

In our series, despite the high incidence of notching (41%), a supracondylar fracture developed in only 1 of 72 patients with a notched femur, which occurred 9 years after surgery. This finding suggests that osseous remodeling probably reduces the risk of fracture in the presence of anterior femoral notch. Sisto et al. (1985) quoted that, in dogs, the bone remodeling around screw holes returns the torsional strength of the bone to normal within 8 weeks of screw removal. Following this observation, Ritter et al. (2005) stated that “this figure is probably too conservative for human application but it is clear that bone remodeling and stress redistribution occur”. Despite the high incidence of substantial anterior femoral notching in our series (41%) and the series published by Ritter et al. (2005) (27%), there was a very low incidence of supracondylar fracture of the femur (0.5%). The latter authors concluded that this femoral defect during TKR is of minimal concern beyond the early postoperative period (0–6 months). Most of our cases had grade I or grade II notching. So with our evidence, minimal notching is not a risk factor for development of supracondylar fractures. We do not have sufficient numbers to comment on grade III and grade IV notching.

In this study, 92 patients had Kinematic condylar knee prostheses, 35 of which had intraoperative notching; 108 patients had Kinemax knee prostheses, 37 of which had intraoperative notching. Of the 35 notched Kinematic condylar knees, 19 patients showed grade I notching, 13 patients had grade II notching, and 3 patients had grade III notching of the femur. However, out of 37 notched Kinemax knees, 20 patients had grade I notching, 15 patients had grade II notching, and 2 patients had grade III notching. This suggests that modification of the jig design to cut the distal femur in the Kinemax knee system does not alter the incidence and degree of notching compared to the Kinematic condylar knee system.

## References

[CIT0001] Aaron RK, Scott R (1987). Supracondylar fracture of the femur after total knee arthroplasty.. Clin Orthop.

[CIT0002] Bogoch E, Hastings D, Gross A, Gschwend N (1988). Supracondylar fractures of the femur adjacent to resurfacing and MacIntosh arthroplasties of the knee in patients with rheumatoid arthritis.. Clin Orthop.

[CIT0003] Booth RE (1994). Management of periprosthetic fractures.. Orthopedics.

[CIT0004] Cain PR, Rubash HE, Wissinger HA, McClain EJ (1986). Periprosthetic femoral fractures following total knee arthroplasty.. Clin Orthop.

[CIT0005] Culp RW, Schmidt RG, Hanks G, Mak A, Esterhai JL, Heppenstall RB (1987). Supracondylar fracture of the femur following prosthetic knee arthroplasty.. Clin Orthop.

[CIT0006] Delport PH, Van Audekercke R, Martens M, Mulier JC (1984). Conservative treatment of ipsilateral supracondylar femoral fracture after total knee arthroplasty.. J Trauma.

[CIT0007] Figgie MP, Goldberg VM, Figgie HE, Sobel M (1990). The results of treatment of supracondylar fracture above total knee arthroplasty.. J Arthroplasty.

[CIT0008] Healy WL, Siliski JM, Incavo SJ (1993). Operative treatment of distal femoral fractures proximal to total knee replacements.. J Bone Joint Surg (Am).

[CIT0009] Hirsh DM, Bhalla S, Roffman M (1981). Supracondylar fracture of the femur following total knee replacement. Report of four cases.. J Bone Joint Surg (Am).

[CIT0010] Huo MH, Sculco TP (1990). Complications in primary total knee arthroplasty.. Orthop Rev.

[CIT0011] Insall J, Insall JN Fractures in the distal femur..

[CIT0012] Landis JR, Koch GG (1977). The measurement of observer agreement for categorical data.. Biometrics.

[CIT0013] Lesh ML, Schneider DJ, Deol G, Davis B, Jacobs CR, Pellegrini VD (2000). The consequences of anterior femoral notching in total knee arthroplasty. A biomechanical study.. J Bone Joint Surg (Am).

[CIT0014] Madsen F, Kjaersgaard-Andersen P, Juhl M, Sneppen O (1989). A custom-made prosthesis for the treatment of supracondylar femoral fractures after total knee arthroplasty: report of four cases.. J Orthop Trauma..

[CIT0015] Rand JA (1994). Supracondylar fracture of the femur associated with polyethylene wear after total knee arthroplasty. A case report.. J Bone Joint Surg (Am).

[CIT0016] Ritter MA, Faris PM, Keating EM (1988). Anterior femoral notching and ipsilateral supracondylar femur fracture in total knee arthroplasty.. J Arthroplasty.

[CIT0017] Ritter MA, Thong AE, Keating EM, Faris PM, Meding JB, Berend ME (2005). The effect of femoral notching during total knee arthroplasty on the prevalence of postoperative femoral fractures and on clinical outcome.. J Bone Joint Surg (Am).

[CIT0018] Scott RD (1988). Anterior femoral notching and ipsilateral supracondylar femur fracture in total knee arthroplasty.. J Arthroplasty.

[CIT0019] Short WH, Hootnick DR, Murray DG (1981). Ipsilateral supracondylar femur fractures following knee arthroplasty.. Clin Orthop.

[CIT0020] Sisto DJ, Lachiewicz PF, Insall JN (1985). Treatment of supracondylar fractures following prosthetic arthroplasty of the knee.. Clin Orthop.

[CIT0021] Webster DA, Murray DG (1985). Complications of variable axis total knee arthroplasty.. Clin Orthop.

[CIT0022] Wiedel J, Hungerford DS, Karckow KA, Kenna RV Management of fractures around total knee replacement.. Total knee arthroplasty: A comprehensive approach.

[CIT0023] Zalzal P, Backstein D, Gross AE, Papini M (2006). Notching of the anterior femoral cortex during total knee arthroplasty characteristics that increase local stresses.. J Arthroplasty.

